# Police-Identified Psychological Distress, Substance Use, and Physical Violence Among Male Intimate Partner Stalkers

**DOI:** 10.1177/0306624X241228977

**Published:** 2024-02-05

**Authors:** Ebonnie Landwehr, Lynne Roberts, David Garratt-Reed, Chloe Maxwell-Smith

**Affiliations:** 1 Curtin University, School of Population Health, Perth, Western Australia, Australia; 2 Behavioural Science & Health Research Group, Curtin University, Perth, Western Australia, Australia

**Keywords:** stalking, drugs, alcohol, mental health, violence

## Abstract

Risk factors for stalking violence are not well understood and few studies have examined psychological distress and substance use specifically. This study aimed to assess whether factors extant in police data could predict severity of stalking violence against intimate partner victims. Western Australia Police Force provided data for 603 men linked to a stalking charge relating to a female intimate partner. Binomial logistic regressions showed police-identified histories of psychological distress and drug use predicted moderate violence, but not severe violence. A co-occurring history of drug use and alcohol use was the strongest predictor of moderate violence (OR = 6.8). These findings suggest accounting for violence severity and substance type when examining stalking violence risk factors. Whether psychological distress and/or substance use were active problems for the men during their stalking behavior is unknown, however the detection of these problems may indicate an unmet need for treatment among this group.

Stalking, commonly defined as a pattern of unwanted and relentless pursuit, is both a public health and a criminal justice problem (Owens, 2016). Police officers responding to stalking incidents face challenges in assessing the risk of harm to the victim due to the complex nature of the behavior (Storey & Hart, 2011). Approximately one third of all stalking situations involve violence and the risk of violence increases with greater closeness between the stalker and their victim (Churcher & Nesca, 2013; McEwan et al., 2007; Thomas et al., 2008). Victims of intimate partner stalkers are at the greatest risk, with an estimated 42% to 81% of these individuals having experienced violence by their perpetrator, (McEwan et al., 2007; Thompson et al., 2013) pointing to these victims as priorities for the development of evidence-based policies (Suzy Lamplugh Trust, 2022; Victorian Law Reform Commission, 2022). Evidence-based policing is underpinned by the available literature, however studies frequently aggregate physical with sexual violence and fail to differentiate violence severity (Churcher & Nesca, 2013; Spitzberg & Cupach, 2007), despite evidence that different risk factors are associated with moderate (e.g., grabbing, slapping) versus severe (e.g., choking, using a knife against someone) intimate partner stalking violence (James & Farnham, 2003; Sheridan & Roberts, 2011; Thompson et al., 2013). Further insight into the nuances of stalker violence would inform police prioritization of risk factors for specific stalking situations.

Intimate partner stalking behavior has been explained from various perspectives, including attachment, relational goal pursuit, and feminist theories (Creamer & Hand, 2022; Spitzberg & Cupach, 2003). Feminist theories extending from intimate partner violence research often posit that men use violence against women as a means to gain and retain control, within the norms of a patriarchal society (Ehrensaft et al., 2004). Although intimate partner stalking and intimate partner violence may be related, they are also distinct phenomena (for a discussion of post-relationship stalking, see Senkans et al., 2021). A proportion of stalking perpetrators have behaved violently towards their partner while in the relationship (Edwards & Gidycz, 2014; Flowers et al., 2020; McEwan et al., 2017) and severe intimate partner violence predicts men stalking their women ex-partners (Senkans et al., 2021). However, a history of intimate partner violence perpetration does not guarantee that stalking will develop after the relationship ends (Senkans et al., 2021). By framing intimate partner stalking as an extension of intimate partner violence, important factors related to intimate partner stalking violence may be neglected. In line with this, a recent critique of the theory underpinning intimate partner stalking violence identified inadequate consideration of stalkers’ reactivity to stress and emotional arousal within observational studies of the behavior (Parkhill et al., 2022). Despite theories highlighting the role of emotion (Spitzberg & Cupach, 2003) and self-regulation (Creamer & Hand, 2022), little research has examined the relationship between an individual’s experience of psychological distress and their perpetration of stalking.

## Psychological Distress as a Risk Factor for Stalking Violence

Psychological distress, an umbrella term encompassing many factors (e.g., emotional dysregulation, stress reactivity, difficulty coping), is associated with low self-esteem and a perceived lack of control (Arvidsdotter et al., 2016). Few studies have considered perpetrators’ experience of psychological distress as a risk factor for intimate partner stalking violence, despite evidence suggesting triggering or stressful events (e.g., learning that an ex-partner has a new romantic interest), jealousy, or relational insecurity are more likely to perpetrate intimate partner stalking violence (Flowers et al., 2020; Groenen & Vervaeke, 2009; Roberts, 2005; Thompson et al., 2013). Furthermore, police officers in the United Kingdom have suggested that stalking perpetration increased during the COVID-19 pandemic due to unprecedented levels of psychological distress and increased substance use (Short et al., 2022). Taken together, psychological distress appears to be important in capturing dynamic risk for stalking violence.

## Substance Use as a Risk Factor for Stalking Violence

Substance abuse is an established predictor of stalking violence (Rosenfeld, 2004), yet it is difficult to establish the relationship for intimate partner stalking violence specifically. Studies often aggregate intimate partner stalkers with other stalkers, and male with female stalkers (Churcher & Nesca, 2013; Roberts, 2005; Rosenfeld, 2004). A meta-analysis examining substance use as a risk factor for intimate partner violence found that there was a stronger link between substance use and violence perpetration among men than among women (Cafferky et al., 2018). A limited body of literature indicates substance abuse predicts men’s stalking violence (McEwan et al., 2007) and intimate partner stalking after a breakup (Ornstein & Rickne, 2013). However, James and Farnham (2003) demonstrated the importance of accounting for violence severity as they found that serious stalking violence was associated with an absence of substance use. Intimate partner stalkers have self-reported using substances as a coping strategy (Flowers et al., 2022). However, stalkers’ self-reported substance use levels are often lower than victims’ reports about perpetrators’ use (Flowers et al., 2020).

Studies examining intimate partner stalking violence and substance abuse within police samples have produced mixed results. Belgian police reports indicated that the risk for stalking violence was 4.7 times greater when the stalker was both an ex-partner and abused substances (Groenen & Vervaeke, 2009), however physical and sexual violence were combined, and severity of violence was not considered. Still, results support suggestions by Thompson et al. (2013), for further research following their finding that illicit drug use increased the risk of severe but not moderate intimate partner stalking violence. In contrast, Bendlin and Sheridan’s (2021) study of Western Australian Police Force family violence incident reports involving intimate partners flagged for stalking behavior found perpetrator substance abuse was unrelated to violence. It is likely that cultural and legislative differences associated with study samples contribute to discrepancies. The extent to which intimate partner stalking violence is associated with dual substance use (e.g., co-occurring drug and alcohol use) is unclear, however polysubstance use appears to be a risk factor for aggression and physical violence (Duke et al., 2018; Steele & Peralta, 2020).

## Practical Application of Police Data

A police sample is appropriate for assessing the relationship between intimate partner stalking violence, psychological distress, and substance use as police data may capture the perpetrators who have not had contact with a mental health service but who have been recorded by police officers as experiencing psychological distress. Police officers are in a unique position to record illegal, unusual, and antisocial behavior as frequent first responders to incidents involving persons affected by psychological distress and/or substance abuse, even in the absence of an offence being committed (for further discussion, see Miles-Johnson & Morgan, 2022). Furthermore, despite evidence that intimate partner stalkers use public mental health services at higher rates than the general public, few appear to engage with these services prior to or during their stalking episode (Albrecht et al., 2022).

Generally, first response police officers identify immediate risk to the victim(s) and use legal sanctions (e.g., protective orders, arrest) to ensure their safety, whereas investigative police officers triage ongoing risk. Police officers providing the initial response to a stalking complaint have limited resources and information to guide their decision making (Groenen & Vervaeke, 2009). Structured assessment tools, such as the Victoria Police Screening Assessment for Family Violence Risk and Ontario Domestic Assault Risk Assessment, effectively support police to identify high risk cases of intimate partner violence (Hilton et al., 2004; Hilton & Eke, 2016; McEwan et al., 2019). These tools often assess perpetrator substance use, and it is possible this factor is also important when police assess risk in intimate partner stalking cases. Groenen (2006, as cited by Groenen & Vervaeke, 2009) found that police officers were better at assessing risk when provided with a model of relevant risk factors developed from police data. While efficient identification of risk at the time of police attendance and ongoing triaging of risk over time may be facilitated by a risk model generated from police data, building such a model first requires identifying relevant police-identifiable risk factors.

## The Current Study

The aim of this study is to identify whether psychological distress and substance use identified among intimate partner stalker men in a police sample are significant predictors of physical stalking violence. The findings will extend knowledge on intimate partner stalking, addressing risk factors for physical violence and providing policing agencies with practical, evidence-based response options. Strategically, this research will inform ongoing policy development, ultimately contributing to improvements in victims’ outcomes. From an operational view, these policy decisions translate to police responses to complaints of stalking, risk identification, and will assist victims and perpetrators to engage in appropriate interventions. This study was guided by the following research questions:

1. Does perpetrator history of police-identified psychological distress, drug use, or alcohol use predict moderate and/or severe intimate partner stalking violence?
2. When controlling for police-identified substance use, does perpetrator history of police-identified psychological distress predict moderate and/or severe intimate partner stalking violence?
3. Does perpetrator history of police-identified polysubstance use predict moderate and/or severe intimate partner stalking violence?

## Method

### Sample

This study was part of a larger project examining intimate partner stalking. Ethical approval was granted by the Curtin University Human Research Ethics Committee (HRE2019-0709). The project sample of interest was men who had been reported to police for pursuing a female intimate partner, who could be linked across several police data files. The Western Australia Police Force (WA Police Force) provided deidentified archival data for all incidents reported to the agency between 1 January 2005 and 31 December 2014 inclusive, with an offence of stalking attached (*N* = 1,826). The Western Australia Criminal Code (1913) defines stalking within two offences under section 338E: pursuing a person or third party with intent to intimidate (1) or pursuing a person or third party in a manner that could be reasonably expected to, and in fact does, intimidate (2). The police data included: a unique identifier for the incident report; the incident report narrative text; the incident report offence(s) information, which included a unique identifier for the stalking victim and their sex; demographic information for the perpetrator, which included their sex and any police-identified indicators of psychological distress and substance use; and family violence incident reports (effective 18 August 2013).

### Dataset Preparation

Each narrative text was read to identify and record the perpetrator-victim relationship, as only incident reports describing dyads involved in an intimate partner relationship were of interest. Dyads were considered intimate partners if they were currently in, or had at some time been a part of, one of the following relationship types: casual/dating, sexual only, defacto, marriage, or extramarital affair. If the narrative did not describe any relationship status it was coded as missing and excluded (*N* = 461). If the relationship described in the narrative was ambiguous it was coded as unclear and excluded (*N* = 56). Narratives that described relationships not of interest to the project (e.g., family, acquaintance, neighbor, colleague, professional, friend, stranger) were excluded (*N* = 534). An unequivocal intimate partner relationship was identified in 774 incidents. Only 1.7% (*N* = 12) of the final sample related to an ongoing relationship on the basis of the information recorded in the narrative. Only incidents involving a male perpetrator and a female victim were retained (*N* = 704). The term sex is used in this paper as it is not clear whether the data accurately reflects individuals’ gender identities. Sex was limited in the data to male and female. The data represented 620 unique perpetrator-victim dyads as 15 perpetrators pursued multiple victims. Data were analyzed at the perpetrator level (*N* = 603).

### Outcome Variables

There were two dichotomous outcome variables: moderate physical violence (1 = moderate violence, 0 = non-violent) and severe physical violence (1 = severe violence, 0 = non-violent). Physical violence was coded with regard to the perpetrator’s actions towards the victim. Coding was achieved in three stages. First, all behaviors described in the narrative text and family violence incident reports were identified for each incident. A diverse range of behaviors was identified including, but not limited to, phone calls, text messages, hacking, revenge pornography, verbal abuse and threats, surveillance and tracking, assault, breach of protective order, strangulation, sexual assault, threats to hurt or kill, prior violence against the victim, prior use of a weapon against the victim, trespass and burglary, and aggression using a vehicle. A random sample of the narratives (*N* = 80) was coded by two researchers to assess interrater agreement, reported using Cohen’s kappa coefficient (κ). Kappa statistics for the behaviors ranged from 0.8 to 1.0, indicating a high level of agreement between the two coders (MacPhail et al., 2016). No new behaviors were identified by the second coder.

Categorizing the severity of identified behaviors was done according to the classification described by Bendlin and Sheridan (2021). Adapting the revised Conflict Tactics Scale 2 (Strauss et al., 1996), Bendlin and Sheridan categorized perpetrators’ behavior as being non-violent, moderately violent (e.g., grabbed, pushed, slapped), or severely violent (e.g., choked, used a weapon). The minority of the behaviors identified in the current study that did not clearly correspond with their classification were added (see Supplemental Appendix A). Two researchers coded each of the identified behaviors to a corresponding severity category. Initially, these coders agreed on the severity of 83.8% of the behaviors however 100% consensus was reached after discussion. Finally, perpetrators were categorized on the basis of the most severe incident they were involved in. If no information was reported about the perpetrator’s behavior (*N* = 4) the incident was coded as non-violent.

### Predictor Variables

The predictor variables were *police-identified history of psychological distress* (1 = identified, 0 = not identified) and *police-identified history of substance use* (3 = dual drug and alcohol use, 2 = drug use without alcohol use, 1 = alcohol use without drug use, 0 = no substance use identified). The predictor variables were representative of the perpetrators’ characteristics and were collated from both the incident data and other police data which could have been provided by the victim, the perpetrator, or otherwise recorded in the police data. Indicators of psychological distress (e.g., depression, suicide attempt, non-specific mental health problems) and substance use (e.g., addiction, intoxication, misuse) were collated from: the incident report main narratives; family violence incident report narratives and risk factor checkboxes (when present); and the perpetrator’s profile information in the police system. Additional indicators of substance use were collated from the perpetrator’s charge history in relation to alcohol-related and drug possession offences. Charges related to possession with intent to sell or supply were excluded as capturing probable personal use was the focus.

### Analytical Strategy

The data were provided to the researchers in Microsoft Word and Excel files. Data was analyzed using Statistical Package for Social Sciences (SPSS) software (version 28.0). Two binomial logistic regression analyses were conducted: one comparing moderate violence with non-violence and the other comparing severe violence with non-violence. Preliminary chi-square analyses were conducted prior to running the regression models. The present study had an adequate sample size (Bujang et al., 2018) and cross-tabulations of the outcome variables by the two predictor variables showed fewer than 20% of the observed or expected cell frequencies fell below five (Tabachnick & Fidell, 2014). All other assumptions were met. The interaction term was included in each model to assess the importance of a combination of psychological distress and substance use. The value for statistical significance was set at 0.025 after applying a Bonferroni correction due to running two independent models. Associations between the predictor variables and outcome variables were expressed as odds ratios (OR) using a 95% Confidence Interval (CI).

## Results

The behavior reported in the police data was categorized as non-violent for most perpetrators (67.5%) but one third were categorized as using moderate or severe violence (18.6% and 13.9%, respectively). Most perpetrators were identified to a single incident and single victim. A police-identified history of psychological distress was identified for just over half of the sample. Police-identified histories of drug use, alcohol use, or dual drug and alcohol use were less common. Descriptive statistics are provided in Tables 1 and 2.

**Table 1.** Descriptive Statistics by Violence Severity.

	Non-violent		Moderate		Severe		Sample total	
	*n*	%	*n*	%	*n*	%	*n*	%
	407	67.5	112	18.6	84	13.9	603	100.0
Number of victims								
One	396	97.3	110	98.2	82	97.6	588	97.5
Two	11	2.7	1	0.9	1	1.2	13	2.2
Three	—	—	1	0.9	1	1.2	2	0.3
Number of incidents								
One	363	89.2	94	83.9	70	83.3	527	87.4
Two	34	8.4	16	14.3	11	13.1	61	10.1
Three	6	1.5	1	0.9	2	2.4	9	1.5
Four	2	0.5	—	—	1	1.0	3	0.5
Five	2	0.5	—	—	—	—	2	0.3
Six	—	—	1	0.9	—	—	1	0.2
Psychological distress								
Identified	198	48.6	72	64.3	54	64.3	324	53.7
Not identified	209	51.4	40	35.7	30	35.7	279	46.3
Substance use								
No substance use	231	56.8	38	33.9	37	44.0	306	50.7
Alcohol use only^a^	20	4.9	5	4.5	6	7.1	31	5.1
Drug use only^b^	133	32.7	54	48.2	33	39.3	220	36.5
Drug and alcohol use	23	5.7	15	13.4	8	9.5	46	7.6

a Alcohol use without drug use. ^b^Drug use without alcohol use.

**Table 2.** Substance Use by Violence Severity and Psychological Distress.

Severity and substance use	PD identified		PD not identified	
	*n*	%	*n*	%
	324	53.73	279	46.27
Non-violent				
No substance use	77	38.9	154	73.7
Alcohol use without drug use	11	5.6	9	4.3
Drug use without alcohol use	92	46.5	41	19.6
Dual drug and alcohol use	18	9.1	5	2.4
Alcohol use with or without drug use	29	14.6	14	6.7
Drug use with or without alcohol use	110	55.6	46	22.0
Moderate violence				
No substance use	20	27.8	18	45.0
Alcohol use without drug use	3	4.2	2	5.0
Drug use without alcohol use	38	52.8	16	40.0
Dual drug and alcohol use	11	15.3	4	10.0
Alcohol use with or without drug use	14	19.4	6	15.0
Drug use with or without alcohol use	49	68.1	20	50.0
Severe violence				
No substance use	19	35.2	18	60.0
Alcohol use without drug use	4	7.4	2	6.7
Drug use without alcohol use	24	44.4	9	30.0
Dual drug and alcohol use	7	13.0	1	3.3
Alcohol use with or without drug use	11	20.4	3	10.0
Drug use with or without alcohol use	31	57.4	10	33.3

*Note*. PD = psychological distress; percentages may not sum to 100% due to rounding.

The logistic regression model for moderate violence versus non-violence was statistically significant, χ^2^(7) = 26.731, *p* < .001. The model explained 8% (Nagelkerke *R*^2^) of the variance in moderate physical violence, correctly classified 61.7% of cases (cut value = .25) and had a sensitivity and specificity of 61.6% and 61.7%, respectively. Police-identified history of psychological distress and police-identified history of substance use were each statistically significant. Perpetrators identified as having a police-identified history of psychological distress were 2.2 [CI = 1.11, 4.44] times more likely than perpetrators without such history to be categorized as moderately violent. Perpetrators identified as having a police-identified history of drug use (without alcohol use) were 3.34 [CI = 1.57, 7.11] times more likely than perpetrators with no police identified history of substance use to be categorized as moderately violent. Perpetrators identified as having a police-identified history of both drug and alcohol use were 6.84 [CI = 1.68, 27.82] times more likely than perpetrators with no police identified history of substance use to be categorized as moderately violent than non-violent. Police-identified history of alcohol use was not significant, nor was the interaction term. The logistic regression model for severe violence versus non-violence was not statistically significant, χ^2^(7) = 10.234, *p* = .176. The binomial logistic regression analyses are presented in Table 3.

**Table 3.** Binomial Regression Models for Moderate and Severe Violence.

	β	*SE*	Wald	*df*	*p*	*OR*	95% CI	
							Lower	Upper
Moderate violence vs. non–violent								
Psych distress	.80	0.35	5.10	1	.024	2.22	1.11	4.44
Substance use^a^			13.92	3	.003			
Alcohol only	.64	0.82	0.61	1	.434	1.90	0.38	9.49
Drugs only	1.21	0.39	9.76	1	.002	3.34	1.57	7.11
Drugs and alcohol	1.92	0.72	7.23	1	.007	6.84	1.68	27.82
Interaction term			2.97	3	.397			
Psych distress × alcohol only	−.59	1.08	0.30	1	.582	0.55	0.07	4.56
Psych distress × drugs only	−.74	0.50	2.21	1	.137	0.48	0.18	1.27
Psych distress × drugs and alcohol	−1.07	0.85	1.58	1	.209	0.34	0.07	1.82
Severe violence vs. non–violent								
Psych distress	.75	0.36	4.38	1	.037	2.11	1.05	4.25
Substance use^a^			2.39	3	.495			
Alcohol only	.64	0.82	0.61	1	.434	1.90	0.38	9.49
Drugs only	.63	0.44	2.01	1	.156	1.88	0.79	4.49
Drugs and alcohol	.54	1.12	0.23	1	.633	1.71	0.19	15.47
Interaction term			1.06	3	.786			
Psych distress × alcohol only	−.26	1.04	0.06	1	.806	0.78	0.10	5.94
Psych distress × drugs only	−.58	0.56	1.05	1	.306	0.56	0.19	1.69
Psych distress × drugs and alcohol	−.08	1.24	<0.01	1	.947	0.92	0.08	10.37

*Note*. OR = odds ratio; CI = confidence interval; psych distress = psychological distress; a reference category is “no substance use identified”; all predictors refer to police-identified histories; moderate violence is compared with non-violent; severe violence is compared with non-violent.

## Discussion

The present study examined cases of intimate partner stalking to determine whether three police-identified risk factors were predictive of male physical stalking violence against a female victim. Consistent with past research, one third of the stalkers were identified as physically violent (Churcher & Nesca, 2013; McEwan et al., 2007) and police-identified histories of psychological distress, drug use, and alcohol use were common. Police-identified history of psychological distress and police-identified history of substance use were significant predictors of moderate stalking violence, but not severe stalking violence. It is important to highlight the fact that 98% of stalking incidents pertained to intimate relationships that had dissolved and as such, these findings may not apply to situations of stalking in ongoing intimate relationships.

The finding that only moderate violence was significantly predicted by a police-identified history of psychological distress or substance use supports the need to account for behavior severity when examining stalking violence risk factors (James & Farnham, 2003; Sheridan & Roberts, 2011; Thompson et al., 2013). Studies exploring risk factors for physical violence within intimate partner stalking have typically aggregated substance types, yet the present study found that police-identified drug use history was positively associated with moderate violence while a police-identified history of alcohol use without drug use was not. Most importantly, a dual police-identified history of both drug use and alcohol use was the strongest predictor of moderate violence. Despite the strong relationship between violence and dual drug and alcohol use emphasized by a recent meta-meta-analysis (Duke et al., 2018), dual use is rarely addressed in the stalking literature. Valuable knowledge about stalking risk factors may be obscured as a consequence of ignoring violence severity (e.g., moderate vs. severe) and substance types (e.g., alcohol use vs. drug use vs. dual use).

The present study uniquely contributes to the literature by addressing perpetrator-specific risk factors relevant for police. Accordingly, the pattern of results perpetuates the mixed findings reported in extant stalking literature addressing violence severity. The positive relationship between moderate violence and a police-identified history of drug use contrasts with previous research (Bendlin & Sheridan, 2021; Thompson et al., 2013) yet the finding that stalking violence was not significantly predicted by a police-identified history of alcohol use is congruent with existing studies (Bendlin & Sheridan, 2021; James & Farnham, 2003; Thompson et al. 2013). The positive relationship between moderate violence and a police-identified history of psychological distress is consistent with findings by Thompson et al. but conflicts with those by Bendlin and Sheridan, who found perpetrator mental health was not predictive of stalking violence severity. The finding that police-identified histories of psychological distress, drug use, and alcohol use were unrelated to severe stalking violence supports the findings of James and Farnham and Bendlin and Sheridan but contrasts with those of Thompson et al. The latter found that the likeliness of severe violence increased when stalkers reported drug use at the time of stalking, experiencing triggering events that could evoke distress, or feeling anger, as compared with stalkers not reporting these factors. Of note, the current findings are also inconsistent with results of a meta-analysis by Spencer and Stith (2020) showing that a history of mental health problems or substance use slightly increased the risk for men perpetrating intimate partner homicide, a form of catastrophic violence that often involves stalking.

Inconsistencies within the stalking violence severity literature is likely attributable to methodological variation. The present and aforementioned studies differ across sample selection (e.g., community, clinical, police), sample composition (e.g., proportion of male vs. female stalkers), stalking definitions (e.g., self-reported, stalking charge), substance use definitions (e.g., use during stalking, static use history), substance types (e.g., drug and alcohol separately vs. aggregated), and indicators of psychological distress (e.g., anger, diagnosis of depression, police-identified history of distress). The discrepant findings demonstrate the importance of definitional consistency in stalking research and indicate heterogeneity in risk factors across groups of stalkers (Fox et al., 2011; McEwan et al., 2021).

This study provides tentative evidence for several police-identifiable risk factors being predictive of moderate physical violence perpetrated by male intimate partner stalkers. These findings require replication, but the present study has practical relevance to developing evidence-based, fit-for-purpose strategies to police intimate partner stalking. This should be a policy priority, given past failures to protect victims have led to policing agencies facing increasing scrutiny over their responses to stalking (Suzy Lamplugh Trust, 2022; Victorian Law Reform Commission, 2022). Policymakers and police management should be aware that police-identified histories of drug use, dual drug and alcohol use, and psychological distress increased the likeliness of moderate physical violence. Likewise, a police-identified history of alcohol use may not be a helpful indicator of risk, as this was not associated with moderate or severe violence. Application of these findings will vary between policing agencies but, overall, are likely to be useful when identifying or triaging risk to inform the most appropriate interventions for a stalking situation.

Police officers commonly perform their duties with limited resources, therefore easily implemented strategies that maximize information may yield improved police responses. Initial attending officers generally focus on ensuring the immediate safety of the involved individuals and the circumstances of a situation may limit the extent to which police data can be easily consulted while responding to an incident. However, supplementing the victim’s report with a simple review of police agency data could identify important risk factors. Compared with victims’ reports of perpetrators’ mental health told to police during interviews (Bendlin & Sheridan, 2021), the present study identified psychological distress more frequently. This aligns with research showing model accuracy for predicting violence improves when existing police data is used to corroborate and supplement victim accounts (Turner et al., 2019).

### Strengths and Limitations

The first strength of this study was the use of naturalistic data. Researchers may find police data to be limited by recording bias, lack of detail, and missing information (Guss et al., 2020) but this is the reality of the data available to police officers responding to incidents. Using an applied approach, this study identified several risk factors that police officers can ascertain by consulting agency data. While the authors recognize potential constraints to accessing data in the field and the varying challenges faced by unique policing jurisdictions, issues of implementation are beyond the present paper. This study extends our understanding of stalkers’ experiences of psychological distress, which has been underexplored thus far (Parkhill et al., 2022).

Several limitations are also acknowledged. First, while psychological distress, drug use, and alcohol use may not explain severe stalking violence, the low proportions of severe violence and police-identified alcohol use identified among males in the sample requires attention. Severe violence may be underreported as perpetrators could have been charged with an offence other than stalking (e.g., assault, homicide). Second, the findings contrast with substantial evidence for alcohol being a strong predictor of physical violence and it is likely that alcohol intoxication is more important than a static history of alcohol use (Duke et al., 2018; Sontate et al., 2021). This is not contradictory to the finding that a police-identified history of drug use predicted moderate violence as these substances are not equivalent. Stalkers who use *illegal* substances may differ from those who do not. Third, psychological distress was analyzed at a binary historical level, and more robust conclusions may be drawn from distress recorded contemporaneously to the stalking episode. Lastly, self-harm and suicidal behavior were coded into psychological distress as it was not possible to determine if threats were help-seeking, manipulative, or indeed both. However, men can use these actions to intentionally cause distress to their ex-partners (refer to Scourfield et al., 2012, p. 471). For some men in the present study, their self-harm or suicidal behavior may have been representative of coercive tactics to control their victim, rather than psychological distress.

### Future Research

Future research should support policing agencies to improve victim experiences by endeavoring to detect risk factors of severe stalking violence that are identifiable by responding officers. Evaluation of the practical application of the risk factors identified in the present study is essential for developing evidence-based and effective policing strategies. Police officers may find it difficult to determine the importance of each identified risk factor, which can interfere with matching appropriate strategies to specific risks (Tayebi & Strand, 2022). This is a considerable gap in knowledge, given that stalking victims often rely on police for advice, and available strategies are not of equivalent effectiveness (Storey & Hart, 2011). Given that severe stalking violence was unrelated to a police-identified drug use history in the present study, it is important to consider implications of police officers’ awareness of links between heavy drug use and dangerous violent offending in the context of intimate partner stalking situations (Kebbell & Westera, 2017).

It is likely that stalkers’ static police-identified histories of psychological distress and substance use can assist police officers during the triaging of stalking incidents. However, exploring motives for substance use, proximity between substance use and stalking behavior, and specific aspects of psychological distress would improve the broader stalking literature. Histories of drug use and/or psychological distress were pervasive among the men in the current study, regardless of physical violence severity, suggesting an unmet need for clinical or community interventions. Notably, stalkers with a police-identified history of drug use were significantly more likely than those without this history to also have a police-identified history of psychological distress (71.4% vs. 39.8%), warranting further exploration.

## Conclusion

This research highlights the value in differentiating physical violence severity when examining stalking violence risk factors. Police-identified histories of psychological distress and drug use were predictive of moderate violence but were not associated with severe violence. A dual history of police-identified drug use and alcohol use was the strongest predictor of moderate stalking violence. As such, police can increase the amount of available information to inform risk triaging by corroborating the material reported by the victim with existing agency data. The evidence relating to the risk factors and processes used by police officers who respond to stalking situations is limited and requires urgent further study.

## Supplemental Material

sj-docx-1-ijo-10.1177_0306624X241228977 – Supplemental material for Police-Identified Psychological Distress, Substance Use, and Physical Violence Among Male Intimate Partner Stalkers Supplemental material, sj-docx-1-ijo-10.1177_0306624X241228977 for Police-Identified Psychological Distress, Substance Use, and Physical Violence Among Male Intimate Partner Stalkers by Ebonnie Landwehr, Lynne Roberts, David Garratt-Reed and Chloe Maxwell-Smith in International Journal of Offender Therapy and Comparative Criminology
