# Supplementary material for: Police-Identified Psychological Distress, Substance Use, and Physical Violence Among Male Intimate Partner Stalkers
Source: Int J Offender Ther Comp Criminol. 2024 Feb 5;69(9):1279–95. doi: 10.1177/0306624X241228977 (PMC12138141; doi:10.1177/0306624X241228977)
Supplement: sj-docx-1-ijo-10.1177_0306624X241228977 – Supplemental material for Police-Identified Psychological Distress, Substance Use, and Physical Violence Among Male Intimate Partner Stalkers [file sj-docx-1-ijo-10.1177_0306624X241228977.docx]

**Appendix**

**Appendix A.**

*Classification of Behaviours – Physical Violence Severity*

| Severity | Behaviour |
| --- | --- |
| Moderate | Threw something at my partner that could hurt  Pulled my partner’s hair  Pushed or shoved my partner  Grabbed my partner  Slapped my partner  Restrained or prevent victim from leaving  Possessed a weapon  Violence/assault not otherwise specified  Spat on victim |
| Severe | Used a weapon on my partner  Punched or hit my partner with something that could hurt  Choked my partner  Threw my partner against something  Beat up my partner or broke a bone  Burned or scalded my partner on purpose  Kicked my partner  Used a vehicle aggressively  Prevented my partner from breathing  Stuck fingers in my partner’s eyes  Bit partner  Pinned my partner down by the throat  Refused my partner medical attention  Solicit to murder my partner  Murdered my partner |
